# Combined systemic and ocular chemotherapy for anterior segment metastasis of systemic mantle cell lymphoma

**DOI:** 10.1186/s12348-015-0060-1

**Published:** 2015-10-08

**Authors:** Aniruddha Agarwal, Mohammad Ali Sadiq, William R. Rhoades, Loren S. Jack, Mostafa Hanout, Philip J. Bierman, William W. West, Quan Dong Nguyen

**Affiliations:** Stanley M. Truhlsen Eye Institute, University of Nebraska Medical Center, 985540 Nebraska Medical Center, Omaha, 68198-5540 NE USA; Division of Hematology and Oncology, Department of Internal Medicine, University of Nebraska Medical Center, Omaha, NE USA; Department of Pathology and Microbiology, University of Nebraska Medical Center, Omaha, NE USA

**Keywords:** Mantle cell lymphoma, Iris, Ibrutinib, Rituximab, Methotrexate, Metastasis, Uveitis, Ultrasound biomicroscopy

## Abstract

**Background:**

Mantle cell lymphoma (MCL) is an aggressive subtype of non-Hodgkin’s lymphoma that rarely metastasizes to the iris and the anterior segment. Blastic/pleomorphic morphology is thought to have an adverse effect on prognosis in MCL. MCL is resistant to conventional chemotherapeutic regimens with a tendency for multiple relapses. Management of anterior segment metastasis of systemic MCL has not been described in literature.

**Findings:**

A 58-year-old male presented with an aggressive, relapsing, metastatic, systemic blastic variant of MCL with ocular involvement. At the time of initial presentation, large tumor cells were visible in the anterior chamber (AC) along with hypopyon and fibrin. The AC cells stained positively for CD20. The iris was thickened and coated with lymphoma cells. Iris neovascularization was present. Given extensive systemic and ocular involvement, the patient was given combination chemotherapy with systemic ibrutinib and intravitreal injections of methotrexate and rituximab. The disease response was monitored using multimodal imaging, including anterior segment optical coherence tomography and ultrasound biomicroscopy. Following combination of systemic and intraocular chemotherapy, there was a marked decrease in the ocular tumor load and the systemic disease.

**Conclusions:**

Combination therapy with intravitreal injections of chemotherapeutic agents targeting monoclonal B-cell population and novel systemic agents may help to achieve remission in anterior segment metastasis of aggressive subtypes of NHL such as blastic variant of MCL. Multimodal imaging may assist in the management of these cases.

## Findings

### Introduction

Mantle cell lymphoma (MCL) is a rare subtype of non-Hodgkin’s lymphoma (NHL) accounting for 6–7 % of all NHL cases. Blastic/pleomorphic morphology is thought to have an adverse effect on prognosis in MCL. MCL is an aggressive form of NHL with poor response to conventional therapy and risk of multiple relapses [1, 2]. Systemic MCL can affect various organs such as lymph nodes, bone marrow, spleen, and bowel resulting in a poor median survival of 3–6 years after diagnosis [3]. However, systemic metastases of NHL to the eye are rare with most cases confined to the orbit and adnexa [4]. Intraocular involvement is even rarer, and it usually affects the choroid [5]. Thus, reports of iris and anterior segment involvement due to metastasis of systemic MCL have been limited to a few case reports [6–8].

While radiation therapy has been used for the management of orbit and adnexal involvement [9], intravitreal injections of chemotherapeutic agents such as methotrexate (MTX) and rituximab (anti-CD20) have been used for patients with vitreoretinal NHL [10, 11]. The treatment for iris metastasis has not been well described in literature. Ibrutinib is a novel Bruton’s tyrosine kinase (BTK) inhibitor recently introduced for the treatment of relapsed systemic MCL [12]. In this index case report, multimodal imaging analysis of a patient with anterior segment metastasis of relapsed systemic MCL is described. Treatment outcome using combination chemotherapy with systemic ibrutinib and intravitreal injections of MTX and rituximab is also discussed.

### Case report

A 58-year-old Caucasian man was diagnosed with systemic MCL, blastic variant, in August 2010. He received six cycles of rituximab hyper-CVAD chemotherapy consisting of alternating combinations of drugs (course A and course B). Course A included cyclophosphamide, vincristine, doxorubicin, and dexamethasone; course B consisted of MTX and cytarabine [13]. Subsequently, the patient received autologous stem cell transplantation (ASCT) with BEAM chemotherapy [bis-chloroethylnitrosourea (carmustine), etoposide, cytarabine, and melphalan] in January 2012 [14]. Following ASCT, the disease was in remission.

A routine restaging positron emission tomography (PET) scan in January 2013 demonstrated relapse of the disease with new, enlarged mediastinal lymph nodes. Biopsy of a gluteal mass revealed metastatic MCL in March 2013. The patient received six cycles of bendamustine plus rituximab salvage [15]. A repeat PET scan in January 2014 did not show any disease activity.

In April 2014, the patient complained of redness, pain, and milky white deposits in the right eye (OD). He was diagnosed with anterior uveitis and elevated intraocular pressure (IOP) and was started on topical prednisone acetate 1 % (four times a day) along with pilocarpine 2 % three times a day. He underwent trabeculectomy and anterior chamber (AC) paracentesis in August 2014. The paracentesis sample was positive for CD20+ lymphoma cells. PET scan demonstrated several areas of nodal and extranodal disease (scalp mass and mediastinal nodes). Cerebrospinal fluid (CSF) and bone marrow biopsy, however, did not reveal lymphoma cells.

**Fig. 1:**
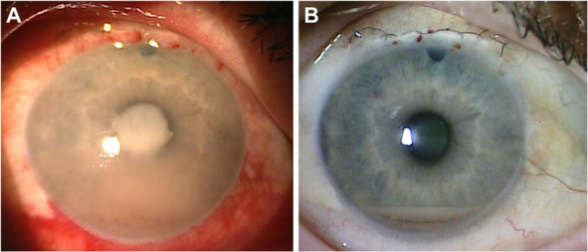
**a** Slit-lamp photograph showing lymphoma involving the iris and anterior chamber (AC). There was diffuse conjunctival injection, a large hypopyon, fibrin over the pupil, and iris neovascularization (especially nasally). **b** Slit-lamp photograph taken 2 weeks after initiation of treatment shows a marked decrease in the conjunctival injection, AC inflammation, and hypopyon. The fibrin is no longer seen. Superior iridectomy and suture from prior trabeculectomy are seen

**Fig. 2:**
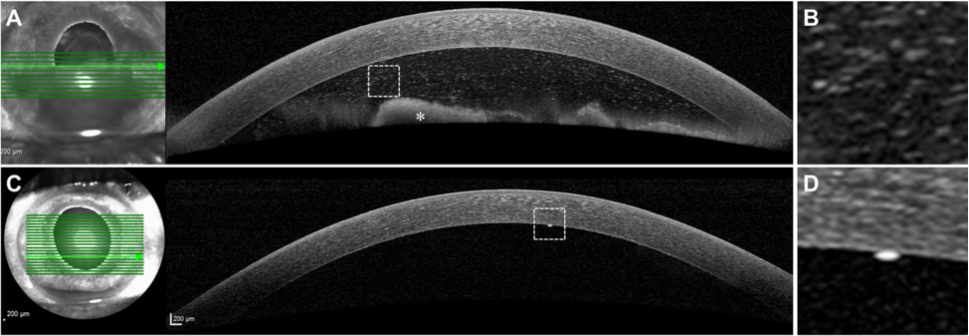
**a** The anterior segment optical coherence tomography (AS-OCT) performed using the cornea protocol shows the presence of a large, irregular hyper-reflective cellular material floating in the anterior chamber (AC). **b** shows the magnified view of the lymphoma cells. **c** The follow-up AS-OCT scan shows a decrease in AC inflammation. **d** A single large granulomatous keratic precipitate is captured and its magnified view is shown

The patient presented to the Stanley M. Truhlsen Eye Institute at the University of Nebraska Medical Center in September 2014 with blurred vision, redness, and pain in OD. Best-corrected visual acuity (BCVA) was counting fingers at 3 m in OD and 20/20 in the left eye (OS). A hypopyon of large tumor cells filled more than half of the AC; large keratic precipitates were seen on the inferior cornea. The iris was thickened and coated with lymphoma cells. There was extensive neovascularization of the iris (NVI). The angles were obliterated and the AC was shallow (Fig. 1a). Anterior segment optical coherence tomography (AS-OCT) (Heidelberg Spectralis®, Heidelberg, Germany) performed using the cornea protocol revealed large cells floating in the AC (Fig. 2a, b). There was marked thickening of the iris of OD compared to OS. Evaluation of the posterior segment with ultrasound B-scan demonstrated a clear vitreous cavity and absence of any retinochoroidal lesions. MRI revealed no orbital, optic nerve, brain parenchymal, or meningeal enhancement.

**Fig. 3:**
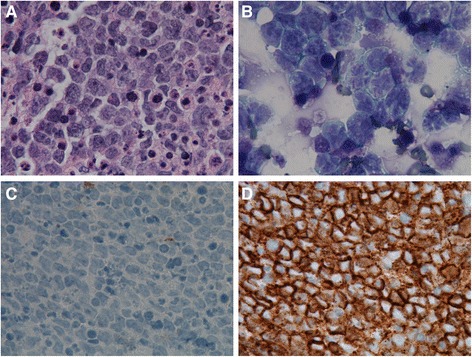
**a** Photomicrograph of cell block showing cytological details of the anterior chamber fluid cells. **a** Hematoxylin and eosin (H&E) stain (100×) demonstrates malignant lymphocytes. **b** Diff-Quik staining (100×) of the sample shows lymphoma cells with atypical, enlarged, irregular nuclei and increased N/C ratio. **c** Immunostaining with CD3 (100×) shows negative staining of the lymphoma cells. **d** Immunostaining with pan-B cell marker CD20 (100×) shows strong membranous staining. The findings on histopathology and the immunoprofile are consistent with the diagnosis of mantle cell lymphoma

**Fig. 4:**
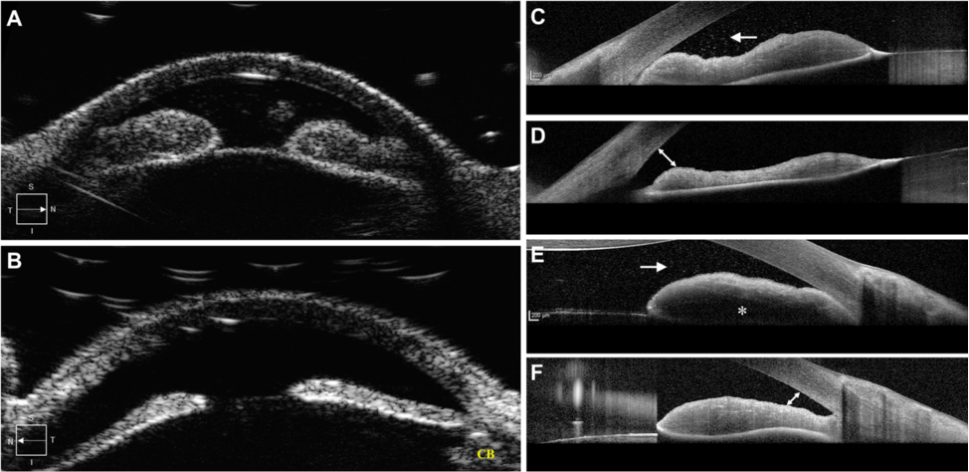
**a** Ultrasound biomicroscopy (UBM) of the right eye shows secondary angle closure due to thickened iris. **b** UBM of the left eye shows normal appearance of the iris of the fellow eye with open angles. *CB* indicates the ciliary body. **c** AS-OCT of the right eye shows the pre-treatment state of the temporal iris. The *white arrow* indicates the floating lymphoma cells. The angle of the *AC* appears closed. **d** The appearance of the iris after institution of therapy reveals a decrease in the thickness and opening up of the angle (*double-sided arrow*). **e** The nasal iris shows partially visible posterior surface of the iris and internal hypo-reflectivity (*asterisk*) due to the increased tissue density anteriorly and poor penetration of the infrared rays through the thick iris. **f** The follow-up scan of the nasal iris after treatment shows reduction in the thickness and clearly visible posterior iris surface. The angle of AC appears open (*double-sided arrow*)

Due to the extensive ocular involvement and the malignant morphology of the CD20-positive cells, combination chemotherapy with intravitreal injections of MTX (400 μg/0.1 ml) and rituximab (1 mg/0.1 ml) were administered on two consecutive days. Intravitreal ranibizumab (0.5 mg) was also administered for the NVI. The oncologist initiated systemic chemotherapy with ibrutinib 560 mg/day. A repeat AC paracentesis was performed for cytological analysis, which revealed monoclonal B-cell lymphoma population positive for CD20, CD5, cyclin D1, and SOX11 but negative for CD10, CD3, and S100 (Fig. 3). Ultrasound biomicroscopy (UBM) (Ellex Eye Cubed™, Adelaide, Australia) findings were consistent with the AS-OCT, showing iris thickening in OD compared to OS (Fig. 4a, b).

At 1-week follow-up, BCVA improved to 20/50 in OD. The hypopyon and iris neovascularization markedly decreased. Evaluation of the posterior segment with fluorescein angiography did not demonstrate any abnormality. During the next 3 months, the patient received two additional injections of intravitreal rituximab at 6-week intervals and one additional injection of MTX and ranibizumab 6 weeks after presentation with interval improvement. AS-OCT demonstrated marked decrease in the AC cells (Fig. 2c). The iris thickening reduced following the combination chemotherapy (Fig. 4c–f). Systemic lymphoma demonstrated stabilization followed by improvement. Ibrutinib therapy was tapered to 420 mg/day due to the concern of secondary myelodysplastic syndrome and an episode of neutropenic fever.

### Discussion

To the best of our knowledge, uveal metastasis of MCL has been reported in only five cases in literature thus far [6–8, 16, 17]. Among these cases, isolated involvement of the anterior segment and iris was observed in two cases [6, 7]. In a report by Economou et al. [6], bilateral involvement of the iris was noted in a 71-year-old male with large, reddish tumor-like masses, anterior chamber inflammation, and hyphema. Reid et al. [7] reported unilateral granulomatous anterior chamber inflammation along with localized undulations of the iris and presence of aberrant-appearing vasculature in an elderly male with systemic MCL. The diagnosis of intraocular MCL was confirmed using cytopathology in only one case by Economou et al. [6]. In all these case reports, the management of secondary iris lymphoma was limited to frequent topical steroids, systemic chemotherapy, or external beam radiation. Since the description of these cases, numerous systemic and local ocular chemotherapeutic strategies have evolved in the management of aggressive relapsing tumors with secondary ocular metastasis. The use of intravitreal chemotherapy along with systemic anti-cancer agents has not been described for the treatment of iris metastasis of MCL or other types of NHL.

Concurrent treatment with intravitreal MTX and rituximab has been successfully employed in the management of vitreoretinal NHL with few adverse events and remission achieved in more than two third of cases [10]. Similarly, for systemic therapy in NHL, rituximab is often combined with other chemotherapeutic agents given its anti-CD20 activity. In this case, we used intravitreal injections of both MTX and rituximab due to the extensive tumor load.

Ibrutinib has been approved by the United States Food and Drug Administration (US-FDA) for the treatment of MCL in patients who have received at least one prior therapy [12]. In our patient, chemotherapy with ibrutinib resulted in marked decrease in the systemic metastatic foci of MCL. However, the exact role of ibrutinib in reducing the ocular tumor load remains unclear.

UBM and AS-OCT complement each other in the diagnosis and management of non-pigmented iris tumors [18]. These techniques produce high-resolution images of the iris tumors resulting in excellent visualization of the tumor anatomy and measurement of tumor dimensions. Careful evaluation of morphological features of the iris on UBM and AS-OCT enabled effective monitoring of the treatment response in our patient (Figs. 2 and 4). To the best of our knowledge, the appearance of lymphoma cells on AS-OCT has not been characterized (Fig. 2). In this case, multimodal imaging allowed an objective assessment of reduction in the tumor load.

### Conclusions

The index case describes successful initial management of anterior segment ocular metastasis in MCL with combined systemic and local ocular chemotherapeutic agents. The treatment regimen may be individualized; results on flow cytometry may guide the selection of appropriate pharmacologic agents after AC paracentesis. Iris metastasis can be well visualized using both UBM and AS-OCT, enabling serial objective assessments of the response to chemotherapy.

### Consent to publish

The consent to publish has been obtained from the participant to report individual patient data.

## Abbreviations

AC: anterior chamber
ASCT: autologous stem cell transplant
AS-OCT: anterior segment optical coherence tomography
BCVA: best-corrected visual acuity
BTK: Bruton’s tyrosine kinase
CSF: cerebrospinal fluid
INV: iris neovascularization
IOP: intraocular pressure
MCL: mantle cell lymphoma
MRI: magnetic resonance imaging
MTX: methotrexate
NHL: non-Hodgkin’s lymphoma
PET: positron emission tomography
UBM: ultrasound biomicroscopy
